# The Functional Role of Zinc Finger E Box-Binding Homeobox 2 (Zeb2) in Promoting Cardiac Fibroblast Activation

**DOI:** 10.3390/ijms19103207

**Published:** 2018-10-17

**Authors:** Fahmida Jahan, Natalie M. Landry, Sunil G. Rattan, Ian M. C. Dixon, Jeffrey T. Wigle

**Affiliations:** 1 Department of Biochemistry and Medical Genetics, University of Manitoba, Winnipeg, MB R3E0J9, Canada; fjaha037@uottawa.ca; 2 Department of Physiology and Pathophysiology, University of Manitoba, Winnipeg, MB R3E0J9, Canada; nlandry@sbrc.ca (N.M.L.); srattan@sbrc.ca (S.G.R.); idixon@sbrc.ca (I.M.C.D.); 3 Institute of Cardiovascular Sciences, St. Boniface Hospital Albrechtsen Research Centre, Max Rady College of Medicine, Rady Faculty of Health Sciences, University of Manitoba, Winnipeg, MB R2H2A6, Canada

**Keywords:** Zeb2, cardiac fibroblast, activated myofibroblast, cardiac fibrosis, fibroblast contractility

## Abstract

Following cardiac injury, fibroblasts are activated and are termed as myofibroblasts, and these cells are key players in extracellular matrix (ECM) remodeling and fibrosis, itself a primary contributor to heart failure. Nutraceuticals have been shown to blunt cardiac fibrosis in both in-vitro and in-vivo studies. However, nutraceuticals have had conflicting results in clinical trials, and there are no effective therapies currently available to specifically target cardiac fibrosis. We have previously shown that expression of the zinc finger E box-binding homeobox 2 (Zeb2) transcription factor increases as fibroblasts are activated. We now show that Zeb2 plays a critical role in fibroblast activation. Zeb2 overexpression in primary rat cardiac fibroblasts is associated with significantly increased expression of embryonic smooth muscle myosin heavy chain (SMemb), ED-A fibronectin and α-smooth muscle actin (α-SMA). We found that Zeb2 was highly expressed in activated myofibroblast nuclei but not in the nuclei of inactive fibroblasts. Moreover, ectopic Zeb2 expression in myofibroblasts resulted in a significantly less migratory phenotype with elevated contractility, which are characteristics of mature myofibroblasts. Knockdown of Zeb2 with siRNA in primary myofibroblasts did not alter the expression of myofibroblast markers, which may indicate that Zeb2 is functionally redundant with other profibrotic transcription factors. These findings add to our understanding of the contribution of Zeb2 to the mechanisms controlling cardiac fibroblast activation.

## 1. Introduction

Cardiac fibroblasts are essential for normal cardiac development, function and tissue homeostasis [1,2]. In addition, cardiac fibroblasts play a vital role in controlling both the inflammatory response and wound healing following cardiac injury [3]. Cardiac fibroblasts are recruited to the damaged myocardium by cytokines and growth factors that are secreted by circulating inflammatory cells [4,5]. As fibroblasts infiltrate the injured area, they undergo rapid proliferation and phenoconversion into myofibroblasts, a key step in fibrogenesis [6,7]. Myofibroblasts remodel the extracellular matrix (ECM) by the de-novo secretion and organization of matrix proteins such as type I and III fibrillar collagens [7,8] and polymerized fibronectin [9]. They also frequently (but not exclusively) express contractile proteins such as α-smooth muscle actin (α-SMA), filamentous actin (F-actin) in stress fibres, and embryonic smooth muscle myosin heavy chain (SMemb), which facilitate contraction and closure of the wound [2,10]. In most cases of wound healing, the new scar is reduced by means of tissue regeneration and the apoptotic removal of myofibroblasts [11]. However, following myocardial infarction (MI), cardiomyocytes do not proliferate significantly and thereby lost myocytes are not replaced. The presence of continued inflammation causes myofibroblasts to persist for many years in the infarct scar [12], although deactivation of activated myofibroblasts in the heart is known to occur [13]. Persistence of the infarct scar is associated with loss of normal ventricular geometry that leads to eventual cardiac dysfunction including impaired inotropic and lusitropic function [14]. Cardiac fibrosis is a primary contributor to other cardiovascular diseases including hypertension, dilated cardiomyopathy and ischemia. Various factors can lead to conversion of cardiac fibroblast to myofibroblast including mechanical stress, hypoxia, signaling ligands such as transforming growth factor-β (TGF-β), connective tissue growth factor (CCN2/CTGF), platelet-derived growth factor (PDGF), angiotensin II, endothelin I, fibroblast growth factor (FGF) and insulin-like growth factor 1 (IGF1) [2,15,16,17,18].

Despite its primary contributions to cardiac dysfunction, there are no effective therapies [2,4]. Common drug classes used to treat cardiovascular disease associated with cardiac fibrosis include statins, angiotensin-converting enzyme inhibition (ACEi) and β-blockade which ameliorate plasma cholesterol levels and hypertension [2,19,20,21]. Nutraceuticals such as resveratrol and omega-3 fatty acids have been shown to reduce cardiac fibrosis in rodent models of hypertension [22,23]. Similarly, lycopene derived from tomato skins has been shown to reduce the expansion of interstitial fibrosis that occurs in rat models of myocardial infarction [24]. At a cellular level, resveratrol and cyanidin-3-glucoside have been shown to blunt fibroblast activation in vitro [25,26]. However, these approaches have not been effective yet clinically, potentially due to an inability to achieve an effective therapeutic dose in patients [27]. Therefore, it is crucial to better understand the molecular mechanisms leading to chronic fibrosis in order to discover new therapeutic agents that will improve outcomes for patients.

We investigated the role of the zinc finger E box-binding homeobox 2 (Zeb2) transcription factor in modulating fibroblast activation. Zeb2 is a member of the zinc finger transcription factor family that plays a crucial role in embryonic development, particularly during epithelial-to-mesenchymal transition (EMT) [28]. Zeb2 has been previously shown to repress Meox2 expression, a homeodomain transcription factor, by binding to its promoter [29]. We have previously reported that Ski (a Smad repressor) can contribute to myofibroblast deactivation by downregulating Zeb2, which in turn causes an upregulation of the transcription factor Meox2 [15]. The potential functional role of Zeb2 in fibroblast activation has not yet been addressed in the literature. Herein we provide new evidence that loss of Zeb2 did not alter the expression of myofibroblast-specific markers. However, ectopic expression of Zeb2 promoted fibroblast activation as demonstrated by the increased expression of myofibroblast markers, increased contractility and decreased migratory ability.

## 2. Results

### 2.1. Subcellular Distribution of Zeb2 during Fibroblast Activation

**Figure 1:**
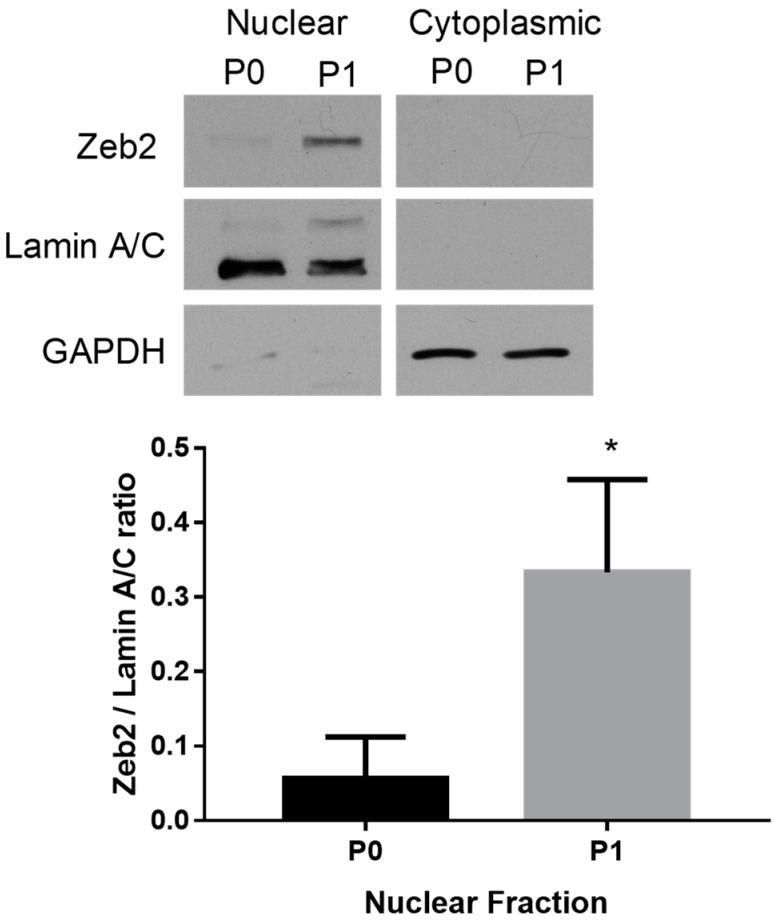
Zeb2 is localized to the nuclei of primary rat cardiac myofibroblasts. Zeb2 protein expression was enriched in the nuclear fraction from P1 rat cardiac myofibroblasts as compared to P0. Lamin and GAPDH were used as nuclear and cytoplasmic loading controls, respectively. The data shown are from *n* = 4 independent experiments, * *p* ≤ 0.05 vs. P0. Error bars represent SEM. Data were analyzed by performing Student’s *t*-test.

To investigate the role of Zeb2 in regulating fibroblast activation, we first compared the subcellular localization of the Zeb2 protein in P0 rat cardiac fibroblasts and P1 myofibroblasts. By Western blot analysis, we determined that the Zeb2 protein was highly expressed in the nuclei of P1 myofibroblasts versus its expression in the nuclei of P0 fibroblasts (*n* = 4, *p* ≤ 0.05) (Figure 1). Zeb2 protein was not detected in the cytoplasmic fractions from either P0 or P1 cells.

### 2.2. The Effect of Zeb2 on the Expression of Myofibroblast Markers

**Figure 2:**
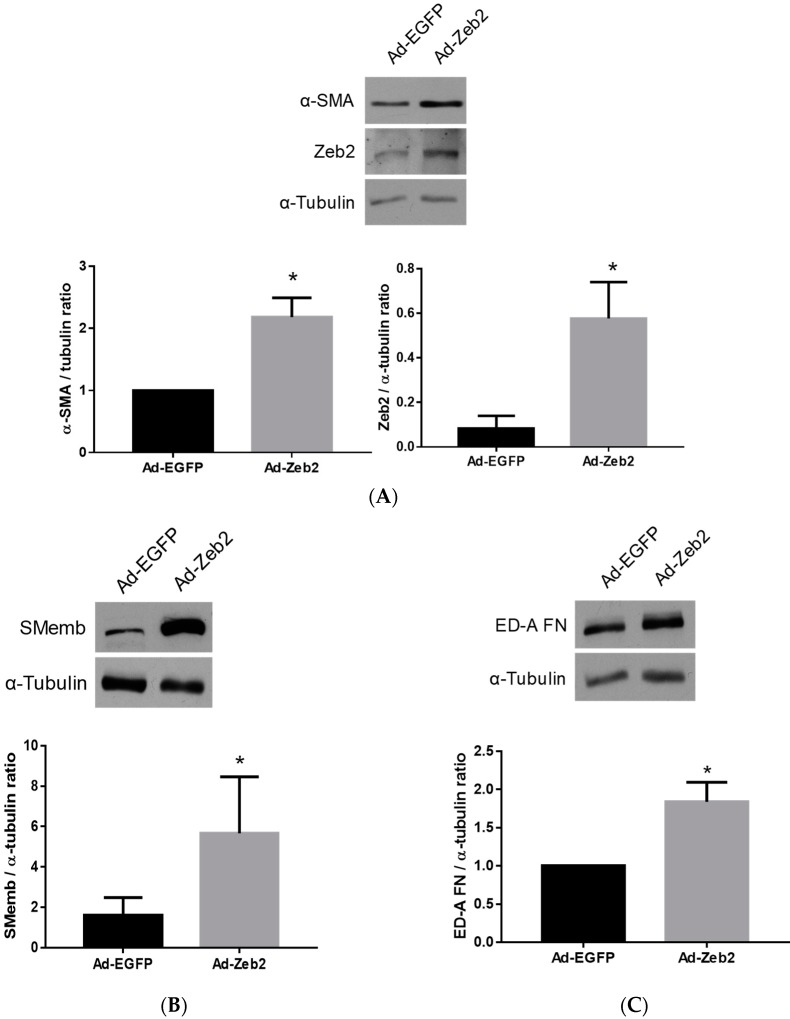
Zeb2 overexpression induces expression of proteins characteristic of the myofibroblast phenotype in primary rat cardiac fibroblasts. P1 cells were transduced with Ad-EGFP or Ad-HA-Zeb2 and incubated for 96 h prior to Western blot analysis. α-Tubulin was used as a loading control. Rabbit polyclonal anti-Zeb2 antibody was used to determine Zeb2 expression levels (Panel A). Ectopic expression of Zeb2 increased the expression of (**A**) α-SMA, (**B**) SMEmb and (**C**) ED-A fibronectin. Right panels are histographic representations of respective expression of markers relative to α-tubulin. The data shown are from *n* = 4 independent experiments for α-SMA and SMemb, and *n* = 3 for ED-A fibronectin. * *p* < 0.05 vs. Ad-EGFP. Error bars represent SEM. Data were analyzed by performing Student’s *t*-tests.

To determine the effects of Zeb2 gain of function on the myofibroblast phenotype, we generated an adenoviral vector encoding Zeb2 (Ad-HA-Zeb2). We were able to achieve a five-fold increase in Zeb2 protein levels by infecting myofibroblasts at a MOI of 200 (Figure 2A). We first examined the effect of ectopic Zeb2 expression on the protein levels of three key markers of the myofibroblast phenotype: α-SMA, SMemb and ED-A fibronectin [28]. Fibroblasts were activated by plating on stiff plastic substrates and transduced with either Ad-EGFP (200 MOI) or Ad-HA-Zeb2 (200 MOI) and incubated for 96 h [29]. Our data shows that Zeb2 overexpression significantly increased the levels of expression of α-SMA (*n* = 4, * *p* ≤ 0.05), SMemb (*n* = 4, * *p* ≤ 0.05) and ED-A fibronectin (*n* = 3, * *p* ≤ 0.05) as compared to the Ad-EGFP infected viral control cells. Expression levels were normalized using α-tubulin as a loading control (Figure 2).

### 2.3. Zeb2 Overexpression Inhibits the Migration and Contractility of P1 Myofibroblasts

Two major functional properties of cardiac myofibroblasts are that (a) they are less motile than fibroblasts, which facilitates their deposition of matrix components within the context of wound healing [28], and (b) they are more contractile than fibroblasts, which allows for the contraction of healing tissue, inherent in wound healing processes in various tissues [4]. Thus, we investigated the effect of Zeb2 on both the migration and contractility of myofibroblasts.

**Figure 3:**
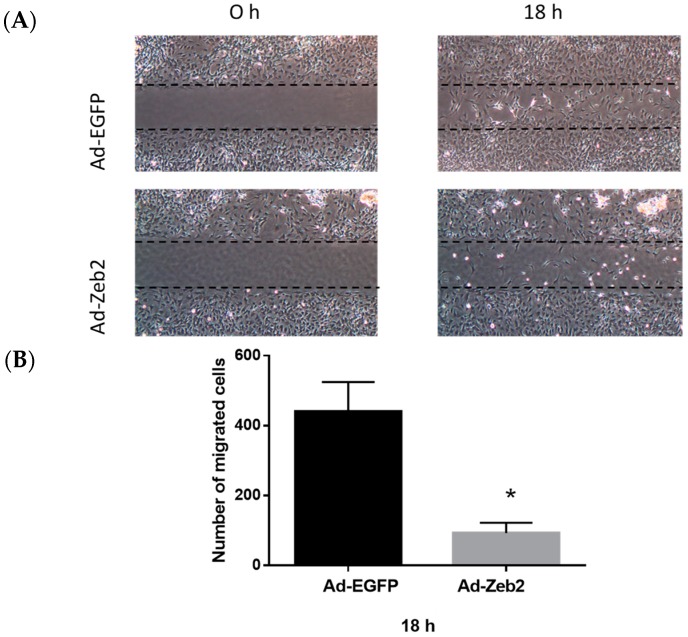
Zeb2 overexpression inhibits the migration of P1 myofibroblasts. (**A**) P1 myofibroblasts were transduced with Ad-EGFP or Ad-HA-Zeb2 and incubated for 96 h. After 96 h, culture inserts were removed and images were taken at the indicated time points (4× objective). (**B**) The number of cells in the wounded area was quantified using ImageJ software. Histographic representation shows the number of migrated cells in Ad-EGFP (200 MOI) and Ad-Zeb2 (200 MOI) infected plates. *n* = 3, * *p* ≤ 0.05 vs Ad-EGFP at 18 h. Error bars represent SEM. Data were analyzed by performing a Student’s *t*-test.

To determine the effect of Zeb2 on cardiac myofibroblast migration, P1 myofibroblasts were transduced with Ad-EGFP or Ad-HA-Zeb2 and incubated for 96 h. After 96 h, culture inserts were removed, medium was changed to 1% FBS-containing medium and images were taken. We found that at 18 h, significantly fewer cells had migrated into the wound area in the Ad-HA-Zeb2 infected plates compared to the Ad-EGFP infected controls (Figure 3).

**Figure 4:**
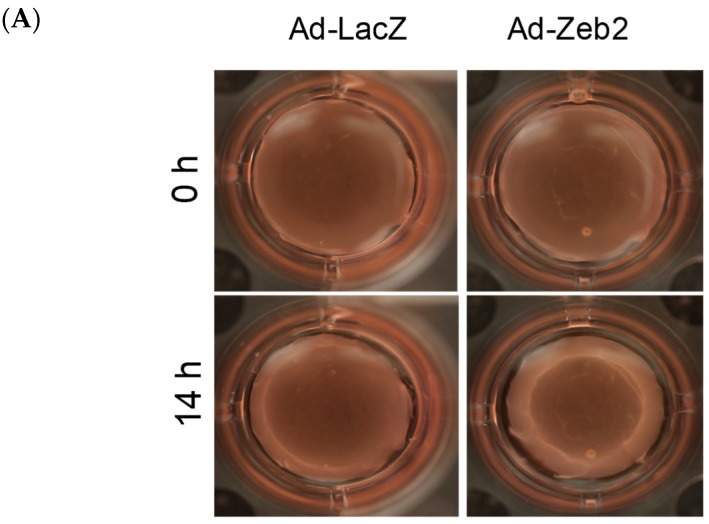

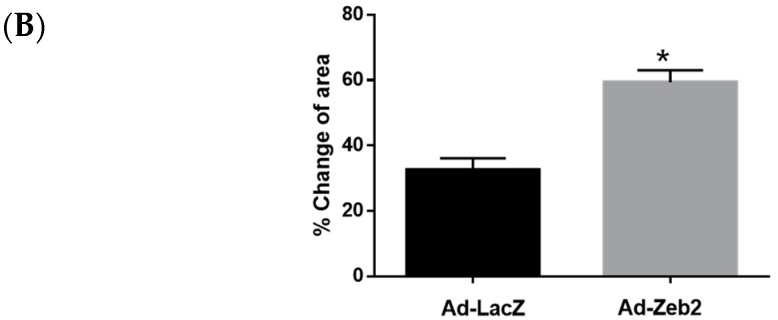
Zeb2 overexpression increases contraction of P1 myofibroblasts—a characteristic of mature myofibroblasts. (**A**) P1 cells were plated on collagen gels and transduced with either Ad-LacZ or Ad-HA-Zeb2 and incubated for 72 h. After 72 h, collagen gels were cut and allowed to contract for 14 h. Images were taken at the 0 h and 14 h timepoints (4× objective). (**B**) The gel size was quantified using Measuregel software. Histographic representation shows the percentage of change of area in case of Zeb2 infected gels compared to Ad-LacZ control. *n* = 3, * *p* ≤ 0.05 vs Ad-LacZ. Error bars represent SEM. Data were analyzed by performing a Student’s *t*-test.

To determine the effect of Zeb2 on myofibroblast contractility, P1 myofibroblasts were plated on collagen gels and transduced with either Ad-LacZ or Ad-HA-Zeb2. After 72 h, gels were detached from the periphery of the plate and the entire gel allowed to “float” for 12 h. Our results show that Zeb2 overexpression increased P1 myofibroblast contractility, as reflected by the increased relative gel contraction versus control gels seeded with Ad-LacZ infected cells, which is indicated by reduced gel size (Figure 4).

### 2.4. Effect of siRNA-Mediated Zeb2 Knockdown on the Expression of Myofibroblast Markers

**Figure 5:**
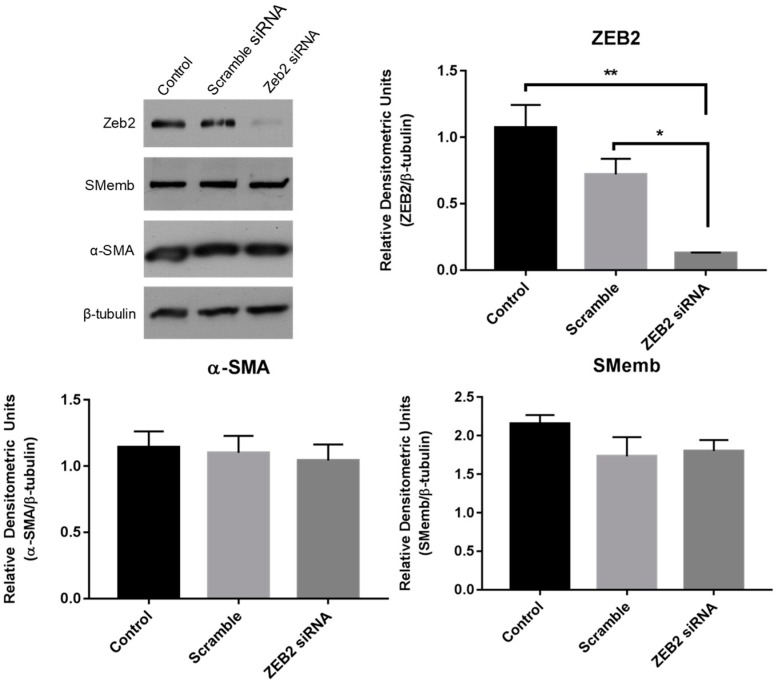
Zeb2 knockdown in primary cardiac myofibroblasts. First-passage (P1) rat cardiac myofibroblasts were subjected to 24 h of serum deprivation prior to treatment with 100 nM of either scramble or FITC-tagged Zeb2-targeted siRNA in serum-free, antibiotic-free DMEM. Untreated cells served as negative controls; transfection efficiency was verified by fluorescent microscopy. Data shown is representative of *n* = 3 biological replicates. ** *p* < 0.01, * *p* < 0.05.

To determine the effect of loss of Zeb2 function on the expression of myofibroblast markers, we knocked down Zeb2 in P1 myofibroblasts and then measured the protein expression of α-SMA and SMemb 24 h later. Transfection with siRNA specific to Zeb2 significantly reduced Zeb2 expression as compared to either control or scramble siRNA controls by 88.1% and 82.3%, respectively (Figure 5). There were no differences detected in the levels of either α-SMA or SMemb between the different groups by Western blot analysis (Figure 5).

## 3. Discussion

In this study, we have demonstrated that Zeb2 regulates cardiac fibroblast activation. Zeb2 overexpression was associated with significant increases in the expression levels of three key myofibroblast markers: α-SMA, SMemb and ED-A fibronectin. Ectopic Zeb2 expression was also associated with reduced myofibroblast migration and a markedly contractile phenotype, which is characteristic of mature myofibroblasts [3,28]. Our study indicates that Zeb2 has profibrotic effects in cardiac fibroblasts, but that its function is not required for maintaining the myofibroblast phenotype.

Previously, we have shown that Ski, a negative regulator of TGF-β signaling, deactivates myofibroblasts by downregulating Zeb2 expression, which in turn is associated with upregulation of its target, the Meox2 homeobox transcription factor. We found that ectopic Meox2 expression led to decreased α-SMA and ED-A FN expression levels [15]. Zeb2 has been previously shown to regulate the TGF-β-mediated EMT process [30]. Moreover, we have observed that Zeb2 expression increases in a post-MI rat model [15]. All of these findings point to a possible role of Zeb2 in regulation of cardiac fibroblast phenotype, and a putative molecular regulatory point for controlling cardiac fibroblast function in chronic remodeling of cardiac matrix deposition by activated fibroblasts.

### 3.1. Zeb2 Regulates Cardiac Myofibroblast Phenotype

Cardiac myofibroblasts characteristically express elevated levels of α-SMA, SMemb and ED-A fibronectin [31,32,33]. Among these markers, SMemb and α-SMA are the two major contractile proteins expressed by myofibroblasts [31,33]. ED-A fibronectin is considered to be one of the major drivers of myofibroblast phenoconversion that plays a role in the induction of α-SMA expression, collagen deposition and cell contractility [32]. We found that infection with adenovirus encoding Zeb2 induces increased expression of these myofibroblast markers (Figure 2). Thus, it is evident that Zeb2 is sufficient to increase the expression of the major myofibroblast markers, and plays a crucial role in driving the phenoconversion of cardiac fibroblasts to myofibroblasts. The increased expression of the myofibroblast markers may result from either increased expression via a Zeb2/SP1-dependent pathway or via Zeb2-mediated repression of an inhibitor of the myofibroblast phenotype either in a SMAD-dependent or -independent manner [30,34,35,36]. Although we have used a relatively high MOI, we have achieved a five-fold induction of Zeb2 expression, which is physiologically relevant (Figure 2A). We have previously shown that Ski overexpression leads to Zeb2 downregulation and that increased Meox2 expression leads to downregulation of α-SMA and ED-A fibronectin protein levels [15]. This finding suggests that an increase in Zeb2 protein levels in cardiac myofibroblasts may lead to downregulation of Meox2 expression, which in turn can increase α-SMA and ED-A fibronectin protein levels. However, in Meox2 overexpression studies, we did not observe a decrease in SMemb expression levels, which suggests that Zeb2 has the potential to regulate myofibroblast phenoconversion either directly or via another pathway [15]. Zeb2 is also known as a Smad-interacting protein, which may reflect Zeb2’s role in fibroblast activation via binding to the Smad transcriptional complex.

### 3.2. Zeb2 Regulates Cardiac Myofibroblast Migration

As myofibroblasts are hypersynthetic, their reduced motility results in increased deposition of new matrix at the precise point of where it may be needed in the course of normal wound healing [2]. Our current results using the wound healing assay show that Zeb2 regulates the migratory properties of cardiac myofibroblasts. Zeb2 overexpression is associated with reduced migration of myofibroblasts, as indicated by the decreased number of cells migrating into the cell-free gap region in the wound healing assay (Figure 4). This result is consistent with the findings of our myofibroblast phenotype marker analysis, where Zeb2 overexpression was shown to induce a hypersynthetic mature myofibroblast phenotype.

### 3.3. Zeb2 Regulates Cardiac Myofibroblast Contraction

Myofibroblasts have a characteristic contractile property that helps in the process of wound closure. Myofibroblasts maintain the matrix in the wound area under tension, which helps reduce wound size and ensures rapid healing [37]. Myofibroblasts synthesize large amounts of contractile proteins such as α-SMA and SMemb, which generate tension by actively contracting to generate force [33,38]. There are also other pathways that can contribute in inducing contractile property of myofibroblasts, for example, Ca^2+^ signaling and Rho/ROCK signaling [39]. We have demonstrated that Zeb2 overexpression in myofibroblasts leads to increased contractility as compared to the Ad-LacZ control (Figure 5). Thus, the finding of increasing myofibroblast marker expression (α-SMA and SMemb) reflects the induction of a more contractile mature cardiac myofibroblast phenotype.

### 3.4. Zeb2 Is Not Required to Maintain the Myofibroblast Phenotype

Fibroblast activation is controlled by a balance between opposing transcription factor signaling pathways. For example, transcription factors such as Scleraxis promote fibroblast activation, whereas transcription factors such as Ski and Meox2 inhibit fibroblast activation [15,40]. We showed that expression of Zeb2 was sufficient to promote fibroblast activation, as shown by expression of markers and functional changes in migration and contractility. However, siRNA-mediated knockdown of Zeb2 had no effect on the expression of two proteins characteristic of the myofibroblast phenotype. This finding may indicate that Zeb2 is not essential for maintenance of the myofibroblast phenotype. Potentially, Zeb1 may compensate for the loss of Zeb2 in maintaining the myofibroblast phenotype [41,42]. Zeb1 and Zeb2 were shown to be targets of miR200 and miR205. Loss of these miRNAs resulted in the upregulation of both Zeb1 and Zeb2 and the subsequent activation of epithelial-to-mesenchymal transition (EMT). The double knockdown of Zeb1 and Zeb2, but not the individual knockdowns, was able to block this activation of EMT [42]. There may exist a similar degree of functional redundancy for these transcription factors in cardiac fibroblasts. Additionally, the knockdown of Zeb2 was effective but there was still 11.9% of Zeb2 protein left that may be sufficient to maintain expression of these markers. Perhaps there is a threshold that needs to be reached before a decrease in Zeb2 is correlated with decreased expression of myofibroblast markers. Finally, the knockdown of Zeb2 was acute (24 h); perhaps an extended knockdown period may result in a phenotypic change. Future studies will be carried out to investigate the importance and existence of these different mechanisms.

Identification of novel regulators of fibrosis for developing selective antifibrotic strategies is currently moving to the mainstream of cardiovascular research. Nutraceutical-based approaches to reduce cardiac fibrosis have shown promise in preclinical models, but more understanding of the underlying mechanisms will improve their clinical effectiveness. Thus, understanding factors that are involved in cardiac fibroblast activation will enable therapeutic targeting to prevent persistent fibroblast activation and progressive fibrosis in chronic stages of heart disease. Overall, the current data supports the hypothesis that Zeb2 promotes cardiac fibroblast activation. We suggest that Zeb2 promotes fibroblast activation, which is indicated by increased expression of myofibroblast markers—the myofibroblastic phenotype is associated with a less motile and more contractile phenotype. Findings from this study directly contribute to our understanding of the biological role of Zeb2 in modulating cardiac fibroblast phenotype, and underscore its putative role in mediating cardiac matrix remodeling.

## 4. Materials and Methods

### 4.1. Cell Isolation and Culture

Approval for experimental protocols for the animal studies was received from the Animal Care Committee of the University of Manitoba, Canada, and the protocols conform to the guidelines established by the Canadian Institutes of Health Research and the Canadian Council on Animal Care (Protocol: 14-049, approved 18 November 2014).

Primary cardiac fibroblasts were isolated from the hearts of adult male Sprague-Dawley rats (150–200 g) [43]. The retrograde Langendorff perfusion method was performed with Dulbucco’s Modified Eagle’s Medium (DMEM)/F12 (Gibco, Thermo Fisher Scientific, Burlington, ON, Canada) followed by Spinner Minimum Essential Medium (SMEM) (Gibco) [43]. After 10 min of perfusion, hearts were digested with 0.1% *w*/*v* collagenase type 2 (Worthington Biochemical Corporation, Lakewood, NJ, USA) in SMEM for 20 min at room temperature. Hearts were then transferred to a 10 cm^2^ plate and the myocardium teased apart in 10 mL of diluted collagenase solution (0.05% *w*/*v*) for 15 min. Then, growth medium (DMEM-F12 supplemented with 10% fetal bovine serum (FBS), 100 U/mL penicillin (Gibco), 100 µg/mL streptomycin (Gibco) and 1 µM ascorbic acid (Sigma-Aldrich, Oakville, ON, Canada)) was added. To remove any large tissue pieces, the crude cell suspension was gently passed through a 40 μm sterile cell strainer (Thermo Fisher) and collected in a 50 mL conical tube which was then subjected to centrifugation at 200× *g* for 7 min. Cell pellets were resuspended in growth medium and plated onto 10 cm^2^ plates. Cells were allowed to adhere for 3 h at 37 °C in a 5% CO_2_ incubator, then washed 2–3 times with 1× phosphate-buffered saline (PBS) prior to replacing the growth medium. The following day, cells were washed with PBS twice and fresh medium was added and cells were allowed to grow for 2–3 days before passaging.

### 4.2. Nuclear/Cytoplasmic Fractionation

P0 cardiac fibroblasts were allowed to grow for 72 h to achieve 70% confluency and were either harvested or passaged to P1 myofibroblasts, which were allowed to grow for 48 h before harvesting. Fractionation was then carried out using the NE-PER Nuclear and Cytoplasmic Extraction Reagents (Pierce Biotechnology, Waltham, MA, USA) as previously described by us [15]. Protein assays were performed using the DC protein assay [44].

### 4.3. Total Cell Lysate Preparation

Following incubation, cells were washed twice with PBS, and RIPA lysis buffer (50 mM, 150 mM NaCl, 1 mM EDTA, 1 mM EGTA, 1% Triton X-100, 1% sodium deoxycholate, 1% SDS, pH 7.4), containing complete^TM^ protease inhibitor cocktail (Roche Life Sciences, Laval, QC, Canada), was added to lyse cells. Cells were then mechanically scraped and the lysates were vortexed once, incubated on ice for 45 min, revortexed and centrifuged in a tabletop centrifuge at 14,000 rpm at 4 °C for 10 min. Supernatants were transferred to new tubes and protein assays were performed using the DC protein assay [44].

### 4.4. Western Blot Analysis

SDS-PAGE of 10–25 µg of protein was performed on either 8% or 10% reducing polyacrylamide gels. Pre-Stained Standard (Bio-Rad Laboratories, Mississauga, ON, Canada) molecular mass markers were used as a standard. Proteins were transferred to a 0.45 µM nitrocellulose membrane (Bio-Rad). Membranes were blocked in 1× TBS containing 5% (*w*/*v*) skim milk powder for 1 h at room temperature with constant shaking. The following primary antibodies were diluted in 1× TBS with 5% skim milk:rabbit polyclonal anti-Zeb2 (1:1000; Sigma), mouse monoclonal anti-α-tubulin (1:5000; Abcam Inc, Toronto, ON, Canada), rabbit polyclonal anti-β-tubulin (1:5000; Abcam), mouse monoclonal anti-α-smooth muscle actin (1:5000; Sigma), mouse monoclonal anti-ED-A fibronectin (1:1000; Millipore, Bedford, MA, USA), mouse monoclonal anti-SMemb (1:1000; Abcam), mouse monoclonal anti-Lamin A+C (Millipore) and mouse monoclonal anti-glyceraldehyde 3-phosphate dehydrogenase (GAPDH; 1:2000; Abcam). Zeb2, Lamin A+C, GAPDH and SMemb antibodies were incubated overnight at 4 °C while α-SMA, ED-A fibronectin and tubulin antibodies were incubated for 1 h at room temperature. Secondary antibodies were horseradish peroxidase (HRP)-conjugated goat anti-rabbit or goat anti-mouse antibodies (Jackson ImmunoResearch Laboratories Inc., West Grove, PA, USA), which were diluted at 1:5000 in 1× TBS containing 5% skim milk and incubated for 1 h at room temperature with constant shaking. Equal protein loading was confirmed using α- and β-tubulin, GAPDH or Lamin A+C. Protein bands were detected using Western Blotting Luminol Reagent (Santa Cruz Biotechnology, Santa Cruz, CA, USA) and images were developed on CL-Xposure blue X-ray films using Flour S Max Multi Imager (Bio-rad, Hercules, CA, USA).

### 4.5. Adenoviral Constructs

The eGFP-expressing control vector (Ad-EGFP) was a gift from Grant Pierce (University of Manitoba) and the LacZ vector (Ad-LacZ) was a gift from Michael Czubryt (University of Manitoba). The HA-tagged human Zeb2 (Ad-HA-Zeb2) virus was constructed using the pAdEasy™ Adenoviral Vector System protocol (Agilent Technologies, Palo Alto, CA, USA). Briefly, adenovirus encoding N-terminal HA-tagged Zeb2 (mouse) was created by excising the Zeb2 cDNA from pcDNA 3.1 (A gift from Anders Lund, University of Copenhagen) and the Zeb2 cDNA was cloned into the pShuttle-CMV vector (Agilent Technologies, Palo Alto, CA, USA). Linearized pShuttle-Zeb2 plasmid DNA and pAdEasy vector were cotransformed into BJ5183-competent *E. coli* cells. Recombined plasmids were amplified in DH5α cells and transfected into HEK293 cells to prepare primary viral stock. Primary stock was then amplified using HEK293 cells and viruses were purified. Finally, Ad-HA-Zeb2 virus titration was performed in HEK293 cells using the Adeno-X™ Rapid Titer Kit (Clontech Laboratories, Mountain View, CA, USA).

### 4.6. Analysis of Myofibroblast Marker Expression Following Adenoviral Infection

For Western blot analysis, 1.4 × 10^5^ P1 rat cardiac myofibroblasts were plated onto 6 cm^2^ plates in 2 mL of 10% FBS-containing DMEM/F-12 medium and infected with either Ad-EGFP or Ad-HA-Zeb2 at a multiplicity of infection (MOI) of 200 and incubated in a 5% CO_2_ incubator at 37 °C. The following day, feeding medium was replaced with 1% FBS containing DMEM F-12 medium and incubated for another 72 h with 5% CO_2_ and subsequently harvested for protein analysis.

### 4.7. Wound Healing Migration Assay

P1 cardiac myofibroblasts (2.5 × 10^4^ cells in 70 µL of 10% FBS-containing DMEM-F12 medium) were plated inside culture inserts (ibidi USA Inc., Madison, WI, USA) and grown overnight in a 5% CO_2_ incubator at 37 °C. The following day, cells were transduced with either Ad-EGFP (200 MOI) or Ad-HA-Zeb2 (200 MOI) and incubated for 96 h. After 96 h, the culture inserts were carefully removed and cells were washed with 1× PBS and the medium was replaced with 1% FBS-containing DMEM-F12 medium. Images were taken at 0 h and 18 h using a 4× objective. The number of cells in the wounded area was quantified using ImageJ software (version 1.45; National Institutes of Health, Bethesda, MD, USA) [45].

### 4.8. Collagen Gel Contraction Assay

To initiate this assay, 7 mL of cold collagen I solution (Worthington) was mixed with 2 mL of 5× culture medium (DMEM F-12 without serum and antibiotics) in a 50 mL centrifuge tube and the pH was kept between 7 and 7.5. The volume was then adjusted to 10 mL with double-distilled water (ddH_2_O). After that, 500 µL of the mixture was added per well of a 24-well plate. Gels were allowed to solidify by incubating at 37 °C in a 5% CO_2_ incubator for minimum 3 h or overnight. P1 myofibroblasts (5 × 10^4^ per well) were then plated onto wells and transduced with either Ad-LacZ (200 MOI) or Ad-HA-Zeb2 (200 MOI) in 10% FBS-containing DMEM-F12 medium. The following day, medium was replaced with 1% FBS-containing DMEM F-12 medium and incubated for another 48 h. After 48 h of incubation, the medium was replaced with serum-free DMEM F-12 medium. Gels were cut around the edges using pipette tips. Images were taken at 0 h and 14 h, and gel surface contraction was measured using IDL Measure Gel software (University of Calgary, Calgary, AB, Canada).

### 4.9. siRNA-Mediated Gene Knockdown

P1 cardiac myofibroblasts were seeded at 8.0 × 10^4^ cells in each well of a 6-well dish. Cells were left to adhere overnight in DMEM:F-12 (1:1) supplemented with 10% FBS and 100 units/mL of penicillin–streptomycin. The cells were then gently washed twice with 1× PBS, then starved overnight in serum-free, antibiotic-free DMEM. The following day, the myofibroblasts were transfected for 24 h with 100 nM of either scrambled Zeb2 siRNA or Zeb2-targeting FITC-tagged siRNA (Table 1) (Sigma-Aldrich, St. Louis, MO, USA) using Lipofectamine RNAiMax (ThermoFisher, Waltham, MA, USA) as per the manufacturer’s protocol; negative control wells were left in DMEM alone. Whole cell lysates were collected and analysed by Western blot.

### 4.10. Statistical Analysis

All experiments were repeated with 3 biological replicates (*n* = 3). For primary cells, different rat hearts were used as a source of primary fibroblasts and each heart equates to *n* = 1 [46]. Student’s *t*-test was used to compare means between two samples (control and experimental groups) and one-way ANOVA followed by a Tukey’s multiple comparison test was used to compare between 3 samples and results, wherein a difference of *p* ≤ 0.05 was considered as statistically significant.

## Figures and Tables

**Table 1:** siRNA sequences.

Oligo ID	siRNA Target	Sequences	Modification
rZeb2 sense	Targets Rat Zeb2 mRNA	[Flc]GCAAGAAAUGUAUUGGUUU[dT][dT]	5′FITC
rZeb2 antisense	Targets Rat Zeb2 mRNA	AAACCAAUACAUUUCUUGC[dT][dT]	None
rZeb2 scramble	Scrambled sense rZEB2 oligo	GUACGUUAAGGUUAGAUAU[dT][dT]	None
rZeb2scramble_as	Scrambled Zeb2 antisense oligo	AUAUCUAACCUUAACGUAC[dT][dT]	None

Flc: Fluorescein label; dT: Deoxythymidine.

## Abbreviations

ECM	extracellular matrix
α-SMA	α-smooth muscle actin
SMemb	embryonic smooth muscle myosin heavy chain
ZEB2	zinc finger E box-binding homeobox 2
